# Supplementary material for: Effects of teriparatide and bisphosphonate on spinal fusion procedure: A systematic review and network meta-analysis
Source: PLoS One. 2020 Sep 1;15(9):e0237566. doi: 10.1371/journal.pone.0237566 (PMC7462270; doi:10.1371/journal.pone.0237566)
Supplement: S1 Appendix — (PDF) [file pone.0237566.s002.pdf]

Effects of Teriparatide and Bisphosphonate on Spinal Fusion Procedure:

A systematic review and network meta-analysis

(Supplementary)

Index of Supplementary

Supplementary File 1. Database and search strategy

Supplementary File 2. Risk of bias

Supplementary File 3. Forest plot of fusion rate

Supplementary File 4. Cumulative probability and SUCRA of fusion rate

Supplementary File 5. Inconsistency test for network meta-analysis of fusion rate

Supplementary File 6. Publication bias in network meta-analysis of fusion rate

Supplementary File 7. Forest plot of ODI

Supplementary File 8. Cumulative probability and SUCRA of ODI

Supplementary File 9. Inconsistency test for network meta-analysis of ODI

Supplementary File 10. Publication bias in network meta-analysis of ODI

Supplementary File 11. Forest plot of adverse event

Supplementary File 12. Cumulative probability and SUCRA of adverse event

Supplementary File 13. Inconsistency test for network meta-analysis of adverse event

Supplementary File 14. Publication bias in network meta-analysis of adverse event

Supplementary 1

Database and search strategy

Supplementary 1
Database and search strategy

Database	Search strategy
Primary search strategy	#1. terrosu<br>#2. forteo<br>#3. teriparatide<br>#4. parathyroid hormone<br>#5. PTh<br>#6. #1 OR #2 OR #3 OR #4 OR #5<br>#7. bonviva<br>#8. alendronate<br>#9. fosamax<br>#10. olpadronate<br>#11. neridronate<br>#12. nericia<br>#13. pamidronate<br>#14. aredia<br>#15. APD<br>#16. zometa<br>#17. avlasta<br>#18. risedronate<br>#19. actonel<br>#20. boneva<br>#21. bisphosphonate<br>#22. ibandronic<br>#23. disambiguation<br>#24. zoledronate<br>#25. #7 OR #8 OR #9 OR #10 OR #11 OR #12 OR #13 OR #14 OR #15 OR #16 OR #17 OR #18 OR #19 OR #20 OR #21 OR #22 OR #23 OR #24<br>#26. spine fusion<br>#27. spinal fusion<br>#28. interbody fusion<br>#29. inter-body fusion<br>#30. spinal arthrodesis<br>#31. spine arthrodesis<br>#32. spondylosyndesis<br>#33. vertebral fusion<br>#34. lumbar fusion<br>#35. thoracic fusion<br>#36. #26 OR #27 OR #28 OR #29 OR #30 OR #31 OR #32 OR #33 OR #34 OR #35<br>#37. #6 OR #25 AND #36

Supplementary 1

Database and search strategy

Database	Search strategy
Embase (n = 231)	('spine fusion'/exp OR 'dorsal spine fusion' OR 'fusion, spine' OR 'spinal fusion' OR 'spine fusion' OR 'spine interbody fusion' OR 'vertebral condensation' OR 'spine arthrodesis'/exp OR 'interbody fusion'/exp OR 'thoracic fusion' OR 'lumbar fusion'/exp OR 'inter-body fusion') AND ('parathyroid hormone[1-34]'/exp OR 'bovine parathyroid hormone [1-34]' OR 'chs 13340' OR 'chs13340' OR 'forsteo' OR 'forteo' OR 'ly 333334' OR 'ly333334' OR 'movymia' OR 'parathar' OR 'parathormone (1-34)' OR 'parathormone 1 34' OR 'parathormone 1-34' OR 'parathormone [1-34]' OR 'parathyroid hormone (1-34)' OR 'parathyroid hormone 1 34' OR 'parathyroid hormone 1-34' OR 'parathyroid hormone [1 34]' OR 'parathyroid hormone [1-34]' OR 'parathyroid hormone [1-34] peptide' OR 'parathyroid hormone[1-34]' OR 'pth [1-34]' OR 'sun e3001' OR 'sune3001' OR 'teriparatide' OR 'teriparatide (rdna origin)' OR 'teriparatide acetate' OR 'teriparatide recombinant human' OR 'terrosa' OR 'bisphosphonic acid derivative'/exp OR 'biphosphonate' OR 'biphosphonates' OR 'bisphosphonate' OR 'bisphosphonate derivative' OR 'bisphosphonates' OR 'bisphosphonic acid derivative' OR 'diphosphonate derivative' OR 'diphosphonate series' OR 'diphosphonates' OR 'diphosphonic acid derivative' OR 'risedronic acid'/exp OR '1 hydroxy 2 (3 pyridinyl) ethylidene 1, 1 bisphosphonic acid' OR '1 hydroxy 2 (3 pyridyl) 1, 1 ethanebisphosphonic acid' OR '1 hydroxy 2 (3 pyridyl) ethylidene 1, 1 bisphosphonate' OR '1 hydroxy 2 (3 pyridyl) ethylidene 1, 1 bisphosphonic acid' OR '2 (3 pyridinyl) 1 hydroxyethylidene 1, 1 bisphosphonic acid' OR 'acrel' OR 'actonel' OR 'actonel once a week' OR 'atelvia' OR 'benet' OR 'ne 58095' OR 'ne58095' OR 'optinate' OR 'ribastamin' OR 'risedronate' OR 'risedronate monosodium hemipentahydrate' OR 'risedronate sodium' OR 'risedronic acid' OR 'ibandronic acid'/exp OR 'olpadronic acid'/exp OR '1 hydroxy 3 dimethylamino 1, 1 propanediphosphonate' OR '1 hydroxy 3 dimethylaminopropane 1, 1 diphosphonate' OR '3 dimethylamino 1 hydroxy 1, 1 propanediphosphonic acid' OR '3 dimethylamino 1 hydroxypropylidene 1, 1 diphosphonate' OR 'hydroxydimethylaminopropylene diphosphonic acid' OR 'ig 8801' OR 'ig8801' OR 'olpadronate' OR 'olpadronate sodium' OR 'olpadronic acid' OR 'olpadronic acid sodium salt' OR 'sodium olpadronate' OR 'zoledronic acid'/exp OR '1 hydroxy 2 (1 imidazolyl) 1, 1 ethanebisphosphonic acid' OR '1 hydroxy 2 (1h imidazol 1 yl) ethylidenebisphosphonic acid' OR '1 hydroxy 2 (imidazol 1 yl) ethylidenebisphosphonic acid' OR 'aclasta' OR 'cgp 42446' OR 'cgp 42446a' OR 'cgp42446' OR 'cgp42446a' OR 'orazol' OR 'reclast' OR 'zol 446' OR 'zol446' OR 'zoledronate' OR 'zoledronate disodium' OR 'zoledronate trisodium' OR 'zoledronic acid' OR 'zoledronic acid disodium salt hydrate' OR 'zoledronic acid hydrate' OR 'zoledronic acid monohydrate' OR 'zomera' OR 'zometa' OR 'alendronic acid'/exp OR '4 amino 1 hydroxy 1, 1 butanebisphosphonic acid' OR '4 amino 1 hydroxy 1, 1 butanediphosphonic acid' OR '4 amino 1 hydroxybutane 1, 1 diphosphonate' OR '4 amino 1 hydroxybutane 1, 1 diphosphonic acid' OR '4 amino 1 hydroxybutylidene 1, 1 bisphosphonate' OR '4 amino 1 hydroxybutylidene 1, 1 bisphosphonic acid' OR '4 amino 1 hydroxybutylidene 1, 1 diphosphonate' OR '4 amino 1 hydroxybutylidene 1, 1 diphosphonic acid' OR 'alenato' OR 'alend' OR 'alendronate' OR 'alendronate sodium' OR 'alendronate sodium trihydrate' OR 'alendronic acid' OR 'alendros' OR 'alovell' OR 'arendal' OR 'bifemelan' OR 'bifosa' OR 'binosto' OR 'bonapex' OR 'defixal' OR 'dronal' OR 'endronax' OR 'eucalen' OR 'fixopan' OR 'fosalan' OR 'fosamax' OR 'fosmin' OR 'fosval' OR 'marvil' OR 'maxibone' OR 'maxibone 70' OR 'mk 0217' OR 'mk 217' OR 'mk0217' OR 'mk217' OR 'neobon' OR 'oncalst' OR 'onclast' OR 'osdron' OR 'osdronat' OR 'oseotenk' OR 'osficar' OR 'oslene' OR 'osteofar' OR 'osteofos' OR 'osteopor' OR 'osteosan' OR 'osteovan' OR 'osticalcin' OR 'porosal' OR 'sodium alendronate' OR 'teiroc' OR 'tibolene' OR 'voroste' OR 'neridronic acid'/exp OR '6 amino 1 hydroxy 1, 1 hexanebisphosphonic acid' OR '6 amino 1 hydroxy 1, 1 hexanediphosphonic acid' OR '6 amino 1 hydroxyhexane 1, 1 diphosphonic acid' OR '6 amino 1 hydroxyhexylidene 1, 1 bisphosphonate' OR '6 amino 1 hydroxyhexylidene 1, 1 bisphosphonic acid' OR '6 amino 1 hydroxyhexylidene 1, 1 diphosphonate' OR '6 amino 1 hydroxyhexylidene 1, 1 diphosphonic acid' OR 'neridronate' OR 'neridronate sodium' OR 'neridronic acid' OR 'nerixia' OR 'pamidronic acid'/exp OR '(3 amino 1 hydroxypropylidene) 1, 1 diphosphonic acid' OR '1 hydroxy 3 aminopropylidene 1, 1 bisphosphonate' OR '3 amino 1 hydroxy 1, 1 propanediphosphonic acid' OR '3 amino 1 hydroxypropane 1, 1 diphosphonate' OR '3 amino 1 hydroxypropane 1, 1 diphosphonic acid' OR '3 amino 1 hydroxypropylidene 1, 1 bisphosphonate' OR '3 amino 1 hydroxypropylidene 1, 1 bisphosphonic acid' OR '3 amino 1 hydroxypropylidene 1, 1 diphosphonate' OR '3 amino 1 hydroxypropylidene 1, 1 diphosphonic acid' OR '3 amino 1 hydroxypropylidine 1, 1 diphosphonate' OR '3 amino 1, 1 diphosphonopropanol' OR '3 aminohydroxypropylidene 1, 1 diphosphonate' OR '3 hydroxy 3, 3 diphosphonopropylamine' OR 'amidronate' OR 'aminohydroxypropanediphosphonic acid' OR 'aminohydroxypropyldiphosphonate' OR 'aminohydroxypropylidene 1, 1 diphosphonate' OR 'aminohydroxypropylidene diphosphonate' OR 'aminohydroxypropylidenebisphonic acid' OR 'aminohydroxypropylidenebisphosphonate' OR 'aminohydroxypropylidenediphosphonate' OR 'aminomux' OR 'apd' OR 'aredia' OR 'aredronet' OR 'cgp 23339' OR 'cgp 23339a' OR 'cgp23339' OR 'cgp23339a' OR 'disodium 3 amino 1 hydroxypropylidene 1, 1 bisphosphonate' OR 'disodium 3 amino 1 hydroxypropylidene 1, 1 diphosphonate' OR 'disodium pamidronate' OR 'ostepam' OR 'pamidrin' OR 'pamidro cell' OR 'pamidro-cell' OR 'pamidrocell' OR 'pamidromyl' OR 'pamidronat' OR 'pamidronate' OR 'pamidronate disodium' OR 'pamidronate disodium novaplus' OR 'pamidronate sodium' OR 'pamidronato' OR 'pamidronic acid' OR 'pamifos' OR 'pamimed' OR 'paminject' OR 'pamipro' OR 'pamired' OR 'pamisol' OR 'pamitor' OR 'panolin' OR 'panorin' OR 'ribodronat' OR 'sodium pamidronate' OR 'texpami')
PubMed (n = 151)	((terrosu OR forteo OR teriparatide OR parathyroid hormone OR PTH) OR (bonviva OR alendronate OR fosamax OR olpadronate OR neridronate OR nericia OR pamidronate OR aredia OR APD OR zometa OR avlasta OR risedronate OR actonel OR boneva OR bisphosphonate OR ibandronic OR disambiguation OR zoledronate)) AND (spine fusion OR spinal fusion OR interbody fusion OR inter-body fusion OR spinal arthrodesis OR spine arthrodesis OR spondylosyndesis OR vertebral fusion OR lumbar fusion OR thoracic fusion)

Supplementary 2
Risk of bias

Supplementary 2
Risk of bias

Risk of Bias Tool for randomized clinical trials our synthesis

Study	1	2	3	4	5	6	7
Jespersen	Low risk	Low risk	Low risk	Low risk	Low risk	Low risk	High risk
Sheng	Unclear	Unclear	High risk	High risk	Low risk	Unclear	Unclear
Ide	Unclear	Unclear	High risk	Low risk	Low risk	Low risk	Low risk
Ebata	Low risk	Low risk	Low risk	Low risk	Low risk	Low risk	High risk
Chen	Unclear	Unclear	Unclear	Unclear	Low risk	Low risk	Low risk
Ohtori	Unclear	Unclear	High risk	High risk	Low risk	Low risk	Low risk
Li	Low risk	Low risk	Low risk	Low risk	Low risk	Low risk	Low risk
Nagahama	Unclear	High risk	Low risk	Low risk	Low risk	Low risk	High risk

1 sequence generation; 2 allocation concealment; 3 blinding of participants and personnel; 4 blinding of outcome assessment; 5 incomplete outcome data; 6 selective reporting; 7 other bias.

Newcastle-Ottawa Quality Assessment Scale for prospective studies in our synthesis

Study	1-1	1-2	1-3	1-4	2	3-1	3-2	3-3
Seki	★	★	★	★	★★	★	★	★
Cho	★	★	★	★	★★	★	★	★
Yagi	★	★	★	★	★★	★	★	★

1-1 Representativeness of the exposed cohort; 1-2 Selection of the non-exposed cohort; 1-3 Ascertainment of exposure; 1-4 Demonstration that outcome of interest was not present at start of study; 2 Comparability of cohorts on the basis of the design or analysis controlled for confounders; 3-1 Assessment of outcome; 3-2 Was follow-up long enough for outcomes to occur; 3-3 Adequacy of follow-up of cohorts.

Supplementary 3 to 6

Outcomes of fusion rate

Supplementary 3

Forest plot of fusion rate

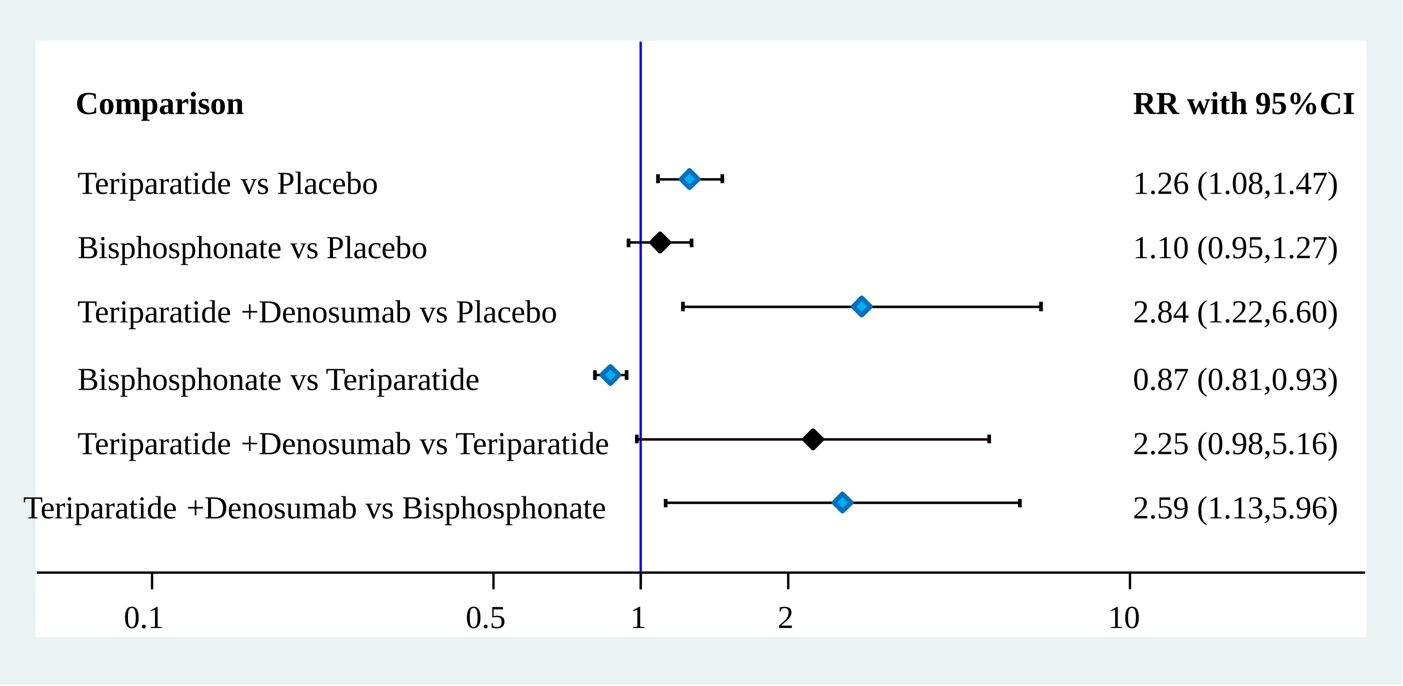

Supplementary 4

Cumulative probability rank and SUCRA of fusion rate

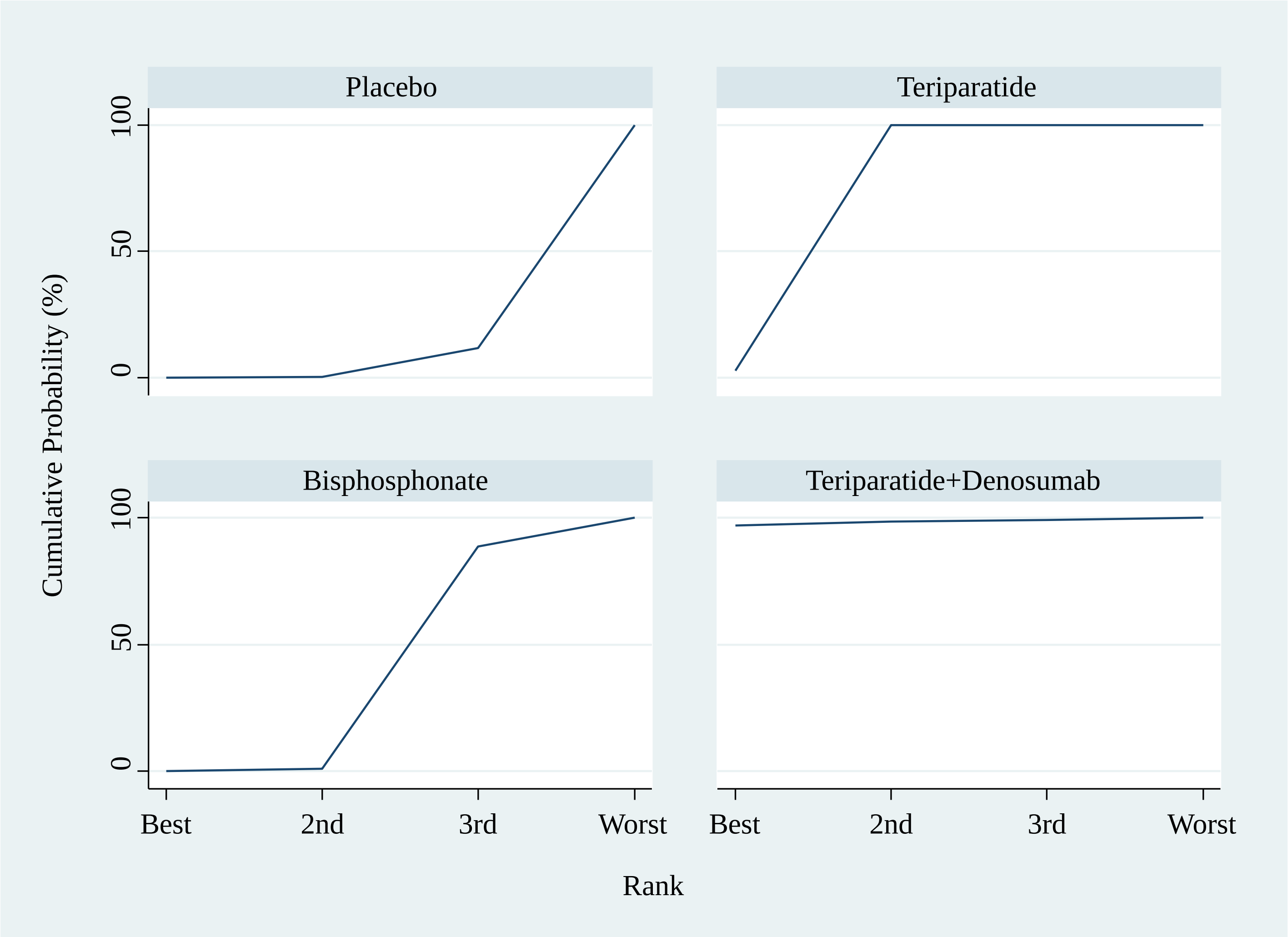

. sucra prob*, labels(Placebo Teriparatide Bisphosphonate Teriparatide+Denosumab)

Treatment	SUCRA	PrBest	MeanRank
Placebo	4.1	0.0	3.9
Teriparatide	67.5	2.8	2.0
Bisphosphonate	30.0	0.0	3.1
Teriparatide+Denosumab	98.4	97.2	1.0

Supplementary 5

Inconsistency test for network meta-analysis of fusion rate

```
. network meta i, luades
Command is: mvmeta _y _S  , bscovariance(exch 0.5)
longparm suppress(uv mm) eq(_y_C: groupB) vars(_y_B _y_C _y_D)

initial:       log likelihood = -18.133634
rescale:       log likelihood = -15.184222
rescale eq:    log likelihood = -13.301489
Iteration 0:   log likelihood = -13.301489
Iteration 1:   log likelihood = -13.097662
Iteration 2:   log likelihood = -13.094284
Iteration 3:   log likelihood = -13.094264
Iteration 4:   log likelihood = -13.094264

Multivariate meta-analysis
Variance-covariance matrix = proportional .5*I(3)+.5*J(3,3,1)
Method = reml                              Number of dimensions     =     3
Restricted log likelihood = -13.094264      Number of observations  =     9
------------------------------------------------------------------------------
             |      Coef.   Std. Err.      z    P>|z|     [95% Conf. Interval]
-------------+----------------------------------------------------------------
_y_B         |
       _cons |   .1492188   .1291635    1.16   0.248    -.103937    .4023746
-------------+----------------------------------------------------------------
_y_C         |
      groupB |  -.1647182    .208889   -0.79   0.430   -.5741332    .2446968
       _cons |    .144175    .103972    1.39   0.166   -.0596065    .3479564
-------------+----------------------------------------------------------------
_y_D         |
       _cons |   .9600011   .4479312    2.14   0.032    .0820721     1.83793
------------------------------------------------------------------------------

Estimated between-studies SDs and correlation matrix:
            SD       _y_B       _y_C       _y_D
_y_B  .0686408          1          .          .
_y_C  .0686408         .5          1          .
_y_D  .0686408         .5         .5          1

Testing for inconsistency:
 ( 1)  [_y_C]groupB = 0

           chi2(  1) =    0.62
         Prob > chi2 =    0.4304
```

Supplementary 6

Publication bias in network meta-analysis of fusion rate

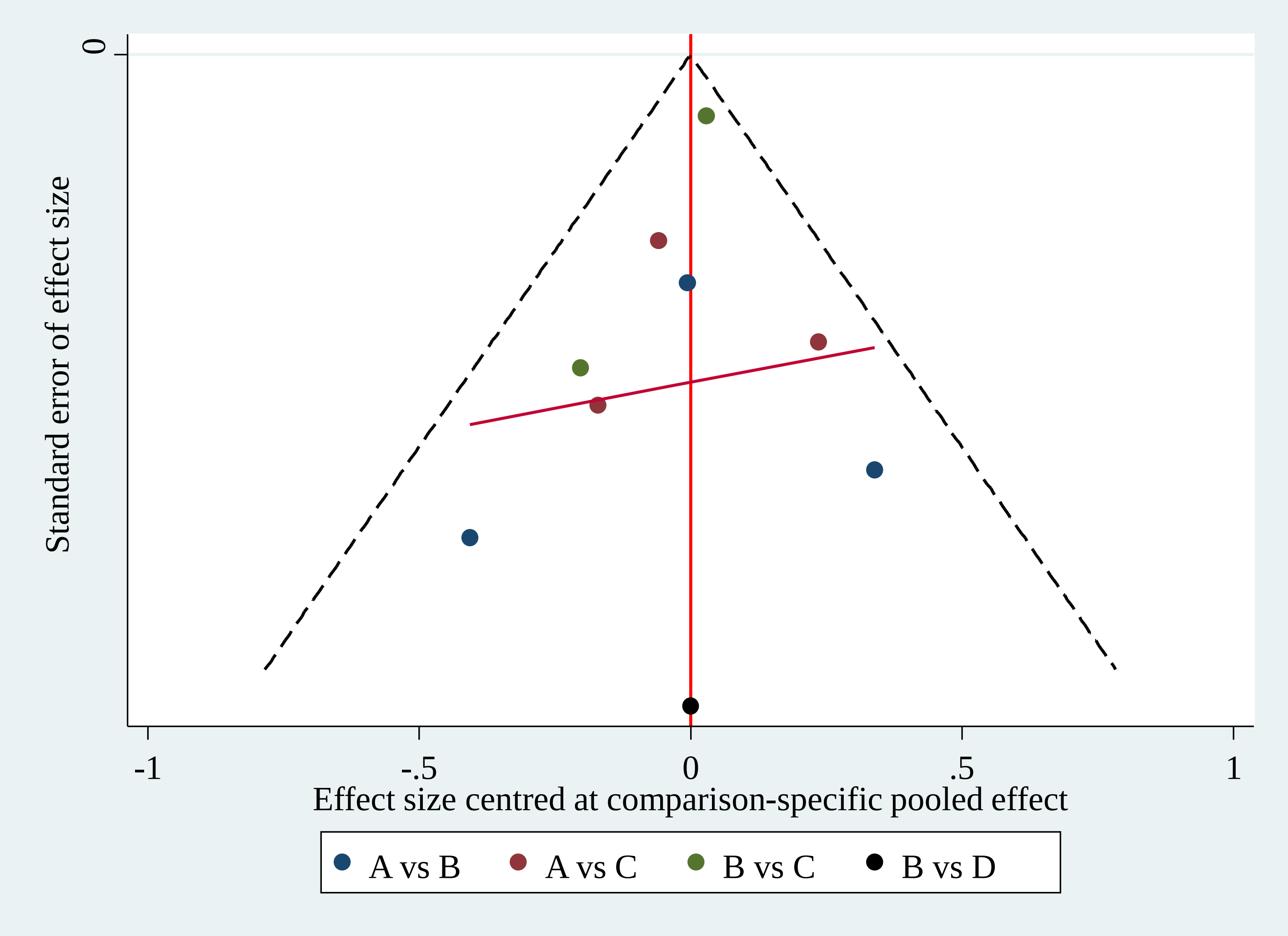

A, Placebo; B, Teriparatide; C, Bisphosphonate; D Teriparatide+Denosumab

Egger's test for small-study effects:
Regress standard normal deviate of intervention
effect estimate against its standard error

Number of studies = 9 Root MSE = .967

Std_Eff	Coef.	Std. Err.	t	P>|t|	[95% Conf. Interval]	
slope	.0390632	.0488657	0.80	0.450	-.0764858	.1546121
bias	-.3122612	.4741488	-0.66	0.531	-1.433445	.8089226

Test of H0: no small-study effects P = 0.531

Supplementary 7 to 10

Outcomes of Oswestry Disability Index (ODI)

Supplementary 7

Forest plot of ODI

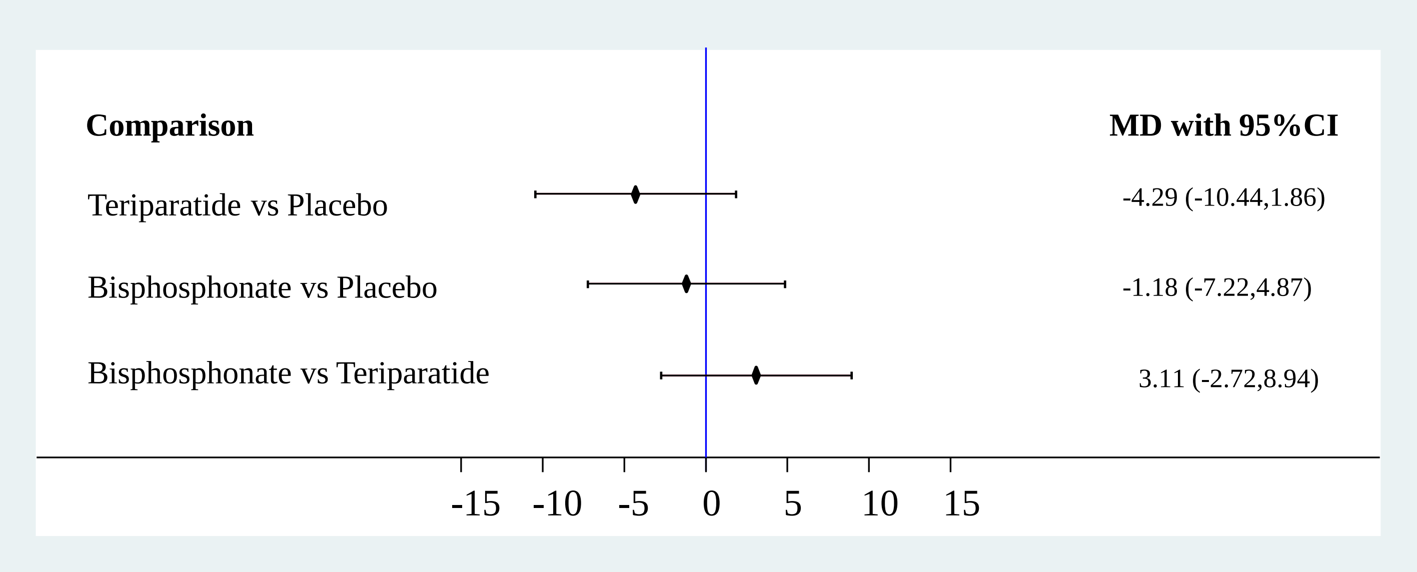

Supplementary 8

Cumulative probability rank and SUCRA of ODI

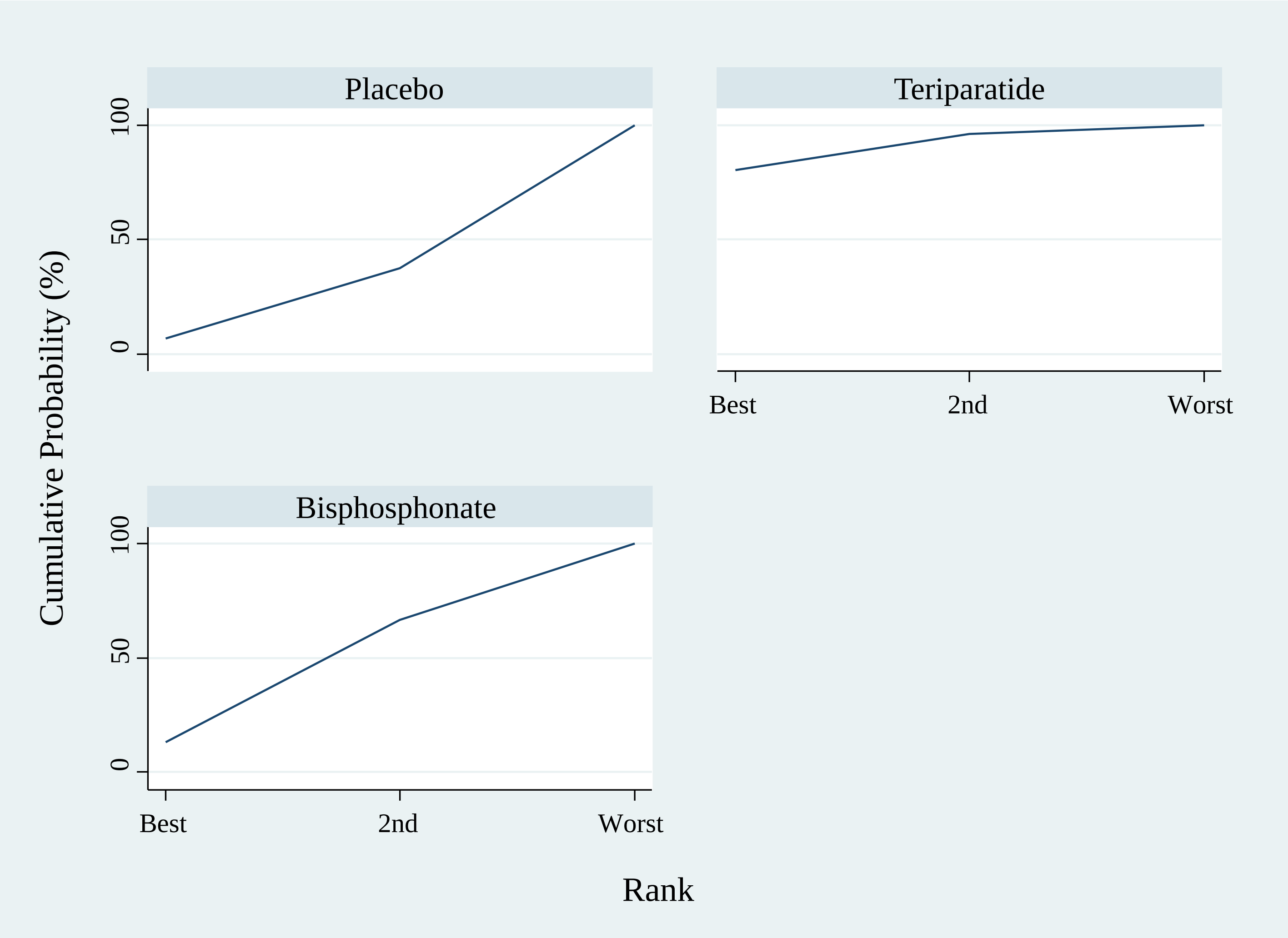

. sucra prob*, labels(Placebo Teriparatide Bisphosphonate)

Treatment	SUCRA	PrBest	MeanRank
Placebo	22.0	6.7	2.6
Teriparatide	88.2	80.4	1.2
Bisphosphonate	39.8	13.0	2.2

Supplementary 9

Inconsistency test for network meta-analysis of ODI

```
. network meta i, luades
Command is: mvmeta _y _S  , bscovariance(exch 0.5) longparm suppress(uv
mm) eq(_y_C: groupB) vars(_y_B _y_C)

initial:       log likelihood = -25.562926
rescale:       log likelihood = -25.562926
rescale eq:    log likelihood = -23.646535
Iteration 0:   log likelihood = -23.646535
Iteration 1:   log likelihood = -23.546692
Iteration 2:   log likelihood = -23.530799
Iteration 3:   log likelihood = -23.530692
Iteration 4:   log likelihood = -23.530692

Multivariate meta-analysis
Variance-covariance matrix = proportional .5*I(2)+.5*J(2,2,1)
Method = reml                                   Number of dimensions    =     2
Restricted log likelihood = -23.530692          Number of observations  =     5
------------------------------------------------------------------------------
             |      Coef.   Std. Err.      z    P>|z|     [95% Conf. Interval]
-------------+----------------------------------------------------------------
_y_B         |
       _cons |  -2.283964   3.020782    -0.76   0.450    -8.204589     3.63666
-------------+----------------------------------------------------------------
_y_C         |
      groupB |   7.696847   5.163088     1.49   0.136     -2.42262    17.81631
       _cons |  -3.022839   2.897123    -1.04   0.297    -8.701095    2.655418
------------------------------------------------------------------------------

Estimated between-studies SDs and correlation matrix:
            SD        _y_B        _y_C
_y_B  3.7862697          1           .
_y_C  3.7862697         .5           1

Testing for inconsistency:
 ( 1)  [_y_C]groupB = 0

           chi2(  1) =    2.22
         Prob > chi2 =    0.1360
```

Supplementary 10

Publication bias in network meta-analysis of ODI

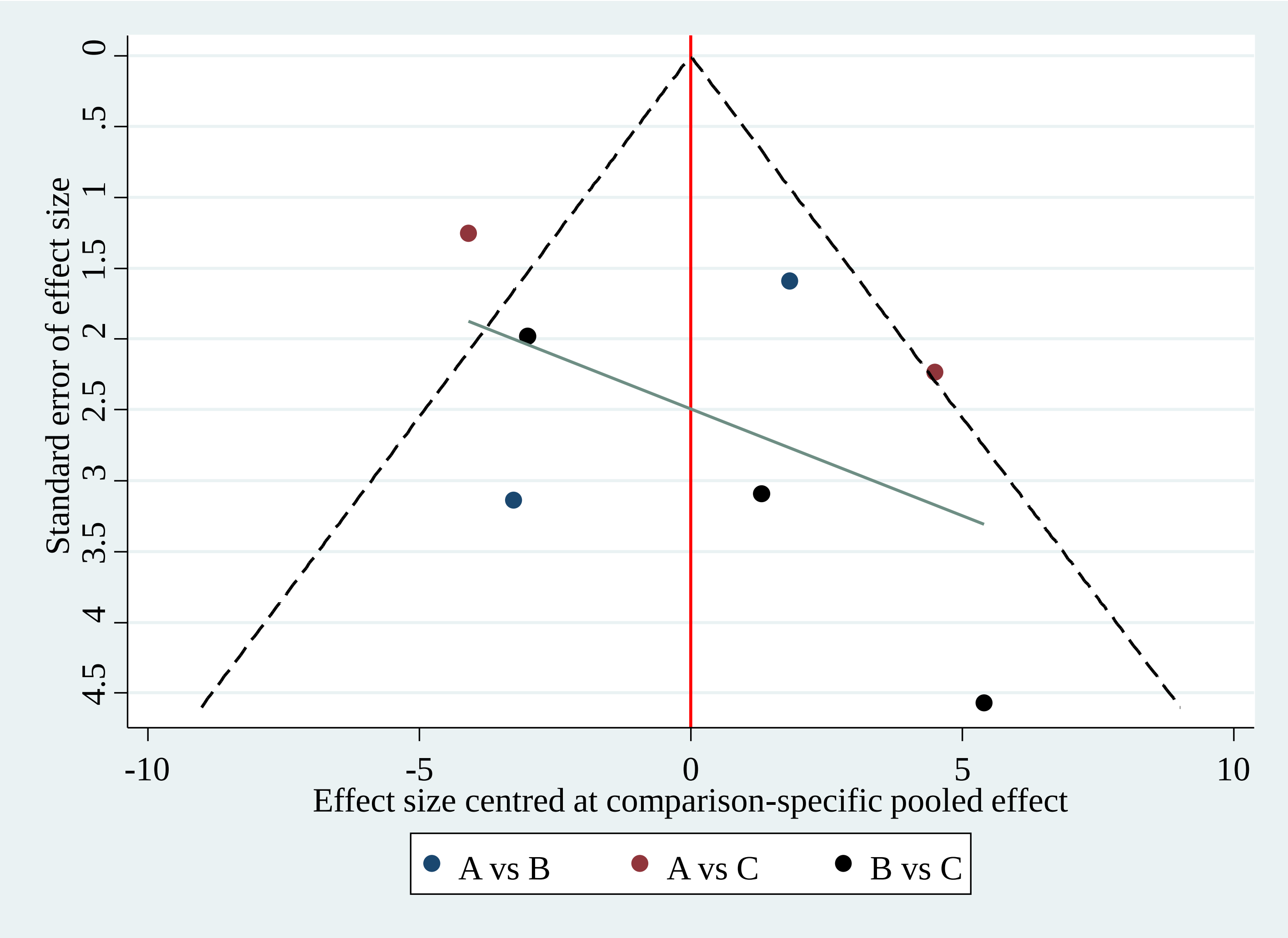

A, Placebo; B, Teriparatide; C, Bisphosphonate

Egger's test for small-study effects:
Regress standard normal deviate of intervention
effect estimate against its standard error

Number of studies = 7 Root MSE = 1.729

Std_Eff	Coef.	Std. Err.	t	P>|t|	[95% Conf. Interval]	
slope	-4.956138	3.513476	-1.41	0.217	-13.98781	4.075539
bias	2.143211	1.752784	1.22	0.276	-2.362464	6.648886

Test of H0: no small-study effects P = 0.531

Supplementary 11 to 14

Outcomes of adverse event

Supplementary 11

Forest plot of adverse event

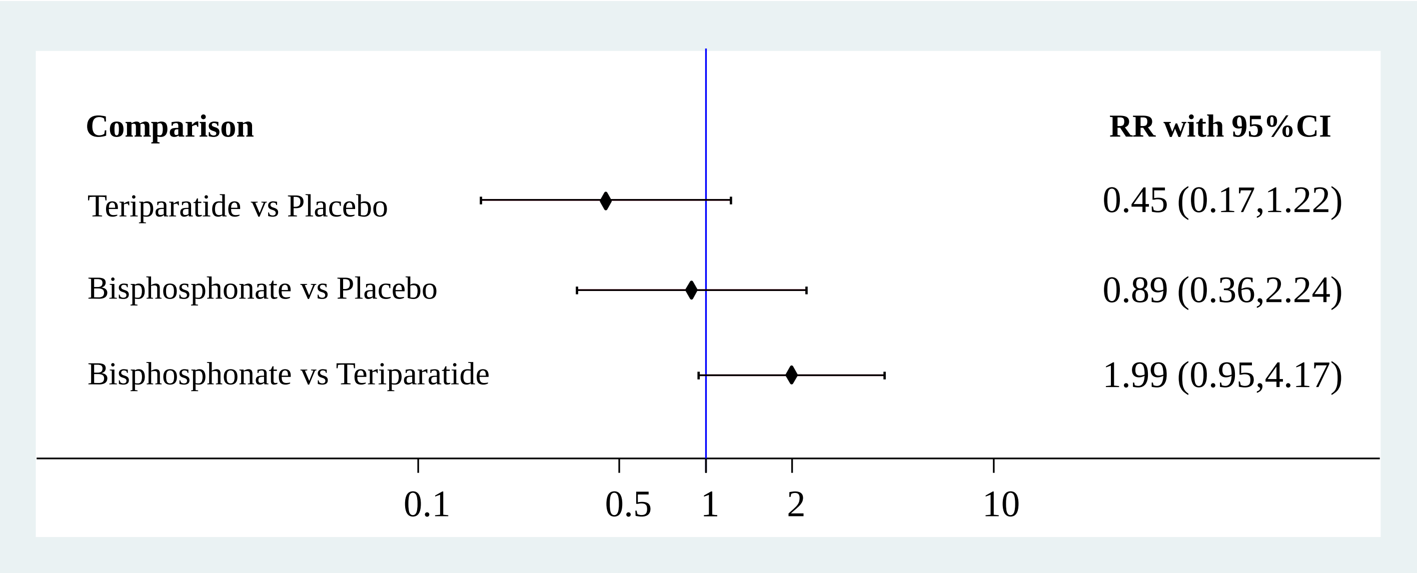

Supplementary 12

Cumulative probability rank and SUCRA of adverse event

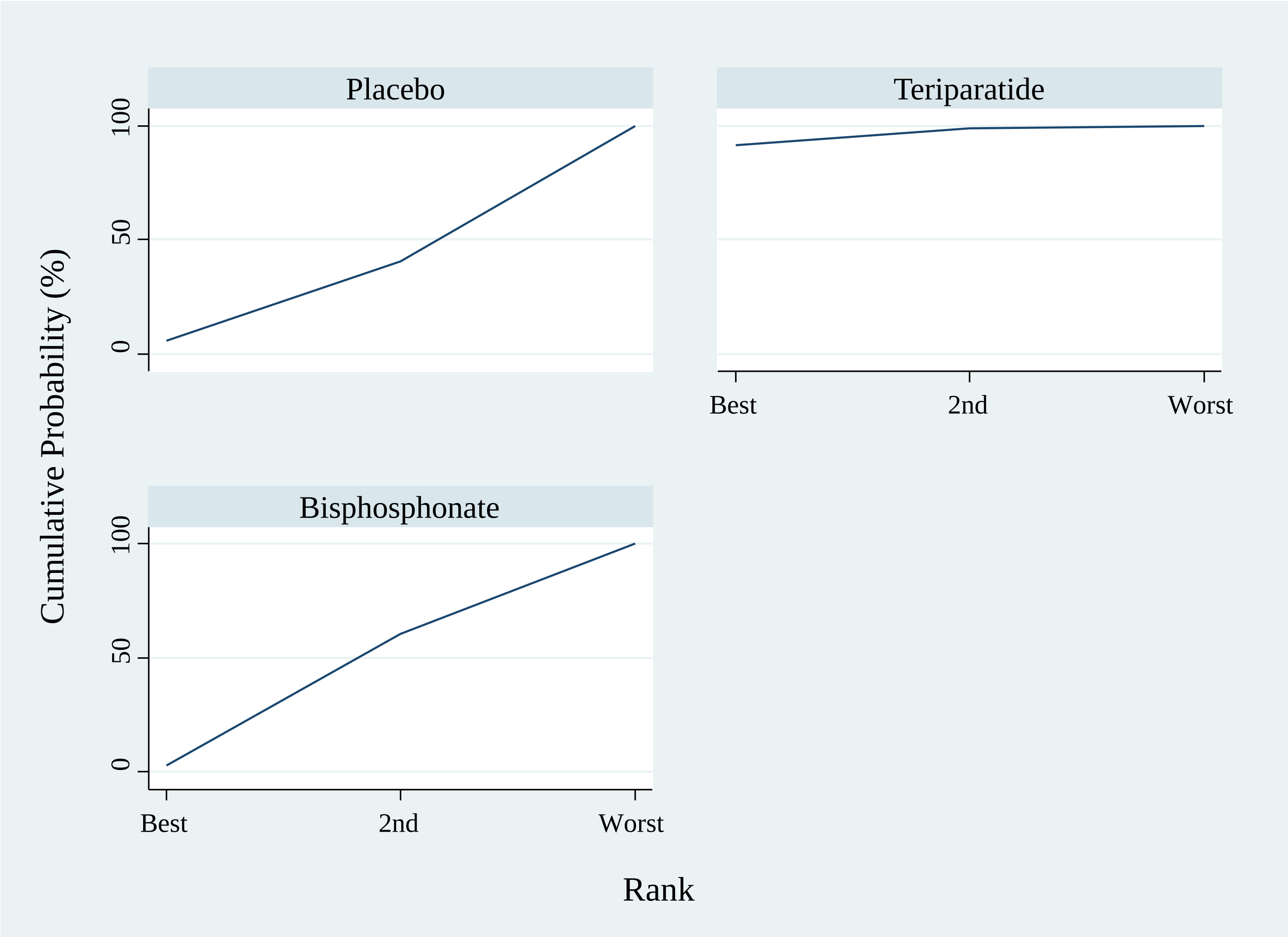

. sucra prob*, labels(Placebo Teriparatide Bisphosphonate)

Treatment	SUCRA	PrBest	MeanRank
Placebo	23.2	5.8	2.5
Teriparatide	95.0	91.2	1.1
Bisphosphonate	31.8	3.0	2.4

Supplementary 13

Inconsistency test for network meta-analysis of adverse event

```
. network meta i, luades
Command is: mvmeta _y _S  , bscovariance(exch 0.5) longparm suppress(uv
mm) eq(_y_C: groupB) vars(_y_B _y_C)

initial:       log likelihood = -13.729116
rescale:       log likelihood = -13.670617
rescale eq:    log likelihood = -13.221721
Iteration 0:   log likelihood = -13.221721
Iteration 1:   log likelihood =  -13.20887
Iteration 2:   log likelihood = -13.208867
Iteration 3:   log likelihood = -13.208867

Multivariate meta-analysis
Variance-covariance matrix = proportional .5*I(2)+.5*J(2,2,1)
Method = reml                              Number of dimensions    =     2
Restricted log likelihood = -13.208867        Number of observations  =     4
------------------------------------------------------------------------------
             |      Coef.   Std. Err.      z    P>|z|     [95% Conf. Interval]
-------------+----------------------------------------------------------------
_y_B         |
       _cons |  -.8913224   .6404926    -1.39   0.164    -2.146665      .36402
-------------+----------------------------------------------------------------
_y_C         |
      groupB |  -.1987626   .8375162    -0.24   0.812    -1.840264    1.442739
       _cons |  -.0632491   .5137735    -0.12   0.902    -1.070227    .9437284
------------------------------------------------------------------------------

Estimated between-studies SDs and correlation matrix:
           SD        _y_B        _y_C
_y_B  1.332e-09         1           .
_y_C  1.332e-09        .5           1

Testing for inconsistency:
 ( 1)  [_y_C]groupB = 0

           chi2(  1) =    0.06
         Prob > chi2 =    0.8124
```

Supplementary 14

Publication bias in network meta-analysis of adverse event

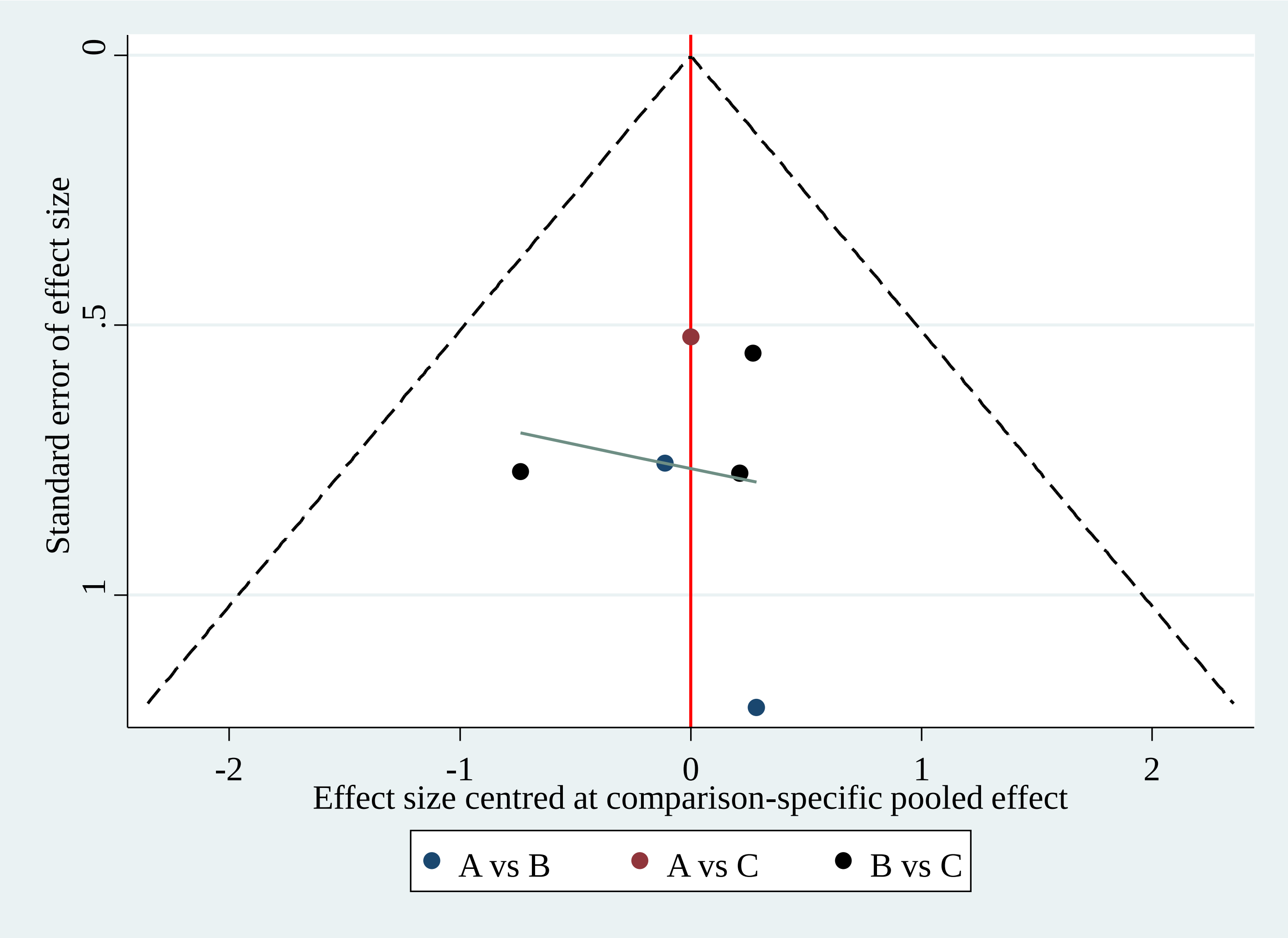

A, Placebo; B, Teriparatide; C, Bisphosphonate

Egger's test for small-study effects:
Regress standard normal deviate of intervention
effect estimate against its standard error

Number of studies = 6 Root MSE = .5658

Std_Eff	Coef.	Std. Err.	t	P>|t|	[95% Conf. Interval]	
slope	.1841819	.6350349	0.29	0.786	-1.578958	1.947321
bias	-.2773287	.9259605	-0.30	0.779	-2.848207	2.29355

Test of H0: no small-study effects P = 0.779
